# Arrhythmic mitral valve prolapse in Kohlschütter–Tönz syndrome

**DOI:** 10.1093/ehjcr/ytaf479

**Published:** 2025-09-20

**Authors:** Annagrazia Cecere, Agatella Barchitta, Lucia Pavan, Martina Perazzolo Marra

**Affiliations:** Department of Cardiac, Thoracic, and Vascular Sciences and Public Health, University of Padova, Via Giustiniani 2, Padova 35128, Italy; Internal Medicine Department, Padova University Hospital, Via Giustiniani 2, Padova 35128, Italy; Medicine Department, Opera Della Provvidenza Sant’Antonio Hospital, Via della Provvidenza, 68, 35030 Sarmeola PD, Italy; Department of Cardiac, Thoracic, and Vascular Sciences and Public Health, University of Padova, Via Giustiniani 2, Padova 35128, Italy

## Case presentation

**Figure 1:**
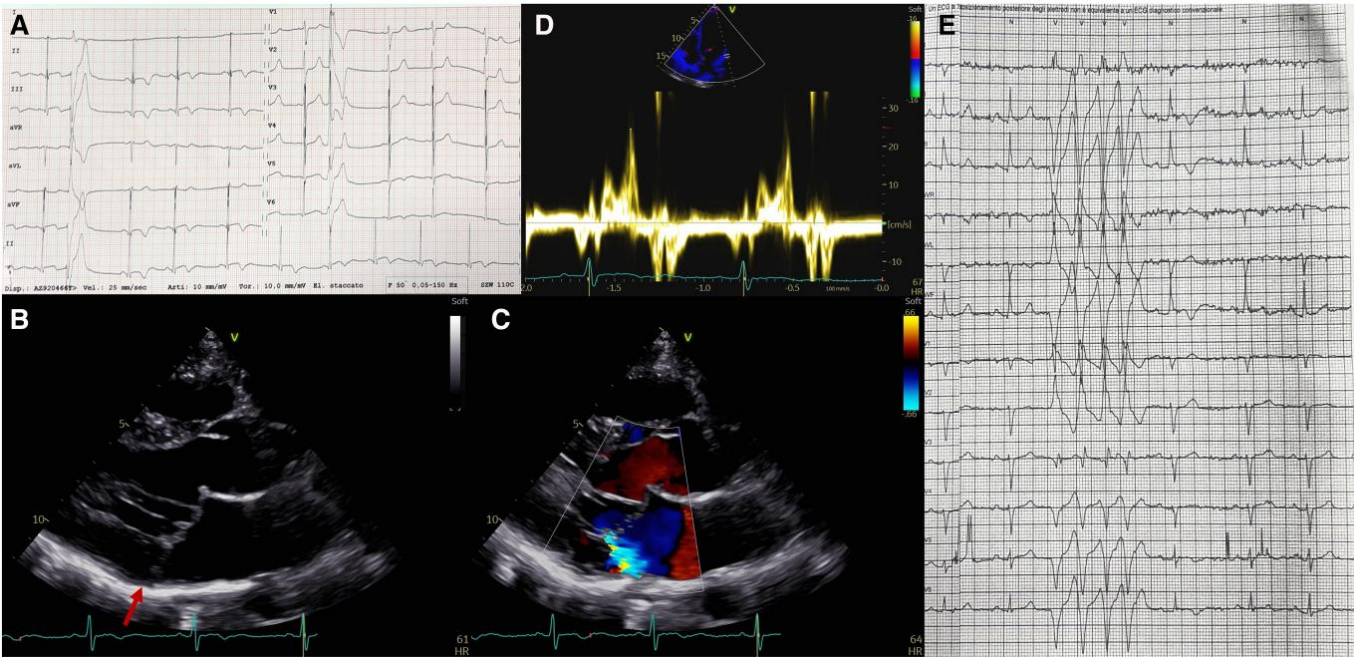
ECG and imaging features of mitral valve prolapse in 41-year-old patient with Kohlschütter–Tönz syndrome. Baseline 12-lead electrocardiogram (*A*) shows sinus rhythm with T-wave inversion in the inferior leads (II, III, aVF) and isolated premature ventricular contraction with right bundle branch block pattern and superior axis morphology. Transthoracic echocardiography showed mitral valve prolapse with inferolateral mitral annular disjunction (red arrow) and mild mitral regurgitation (*B–C*). Tissue Doppler imaging of the lateral mitral annulus showing a Pickelhaube sign (S′ velocity 25 cm/s) (*D*). A 24-h 12-lead Holter monitoring revealed a non-sustained monomorphic ventricular tachycardia with right bundle branch block and superior axis morphology.

A 41-year-old man with genetically confirmed Kohlschütter–Tönz syndrome (KTS) was referred for evaluation of a suspected papillary muscle rupture in the context of mitral valve prolapse (MVP). He had no personal or family history of cardiovascular disease or sudden cardiac death. The patient was institutionalized for drug-resistant epilepsy and behavioural disturbances. Cardiac auscultation revealed a mid-systolic click and apical late systolic murmur. A 12-lead electrocardiogram (ECG) showed sinus rhythm, T-wave inversion in the inferior leads, and isolated premature ventricular contractions (PVCs) with right bundle branch block (RBBB) morphology and superior axis (*Figure 1A*). Transthoracic echocardiography revealed classic MVP with myxomatous thickening of the leaflets, inferolateral mitral annular disjunction, systolic curling, and mild mitral regurgitation^1^ (*Figure 1B* and *C*). Ventricular volumes and function were preserved, without evidence of papillary muscle rupture. A Pickelhaube sign was noted (*Figure 1D*). A 24-h Holter ECG confirmed frequent monomorphic PVCs with RBBB and superior axis (*Figure 1E*). The patient received bisoprolol 2.5 mg and ramipril 1.25 mg once daily. At 2-month follow-up, Holter monitoring showed a reduction in PVC burden. Cardiac magnetic resonance was not performed due to poor compliance. His 27-year-old sister, also affected by KTS, showed no cardiac abnormalities.

First described in 1974, KTS is a rare autosomal recessive disorder caused by biallelic pathogenic variants in *ROGDI* gene.^2^ The hallmark features include refractory epilepsy, psychomotor and intellectual impairment, and hypoplastic amelogenesis imperfecta. Prognosis has been poor due to epilepsy-related complications.^3^ Although *ROGDI* is expressed in the brain, spinal cord, blood, bone marrow, and heart with clinical variability, cardiac involvement has not been previously described.^2^

To our knowledge, this is the first reported case of arrhythmic MVP in a patient with KTS. Although the coexistence may be coincidental, it highlights the need for careful cardiac assessment in KTS patients, particularly when arrhythmogenic features are present.

## Data Availability

The data underlying this article are available in the article.

## Notes

**Consent:** Informed consent for publication of this anonymized case report was obtained from the patient’s legal representative, in compliance with Committee on Publication Ethics (COPE) best practice guidelines.
